# Identification of novel DNA-damage tolerance genes reveals regulation of translesion DNA synthesis by nucleophosmin

**DOI:** 10.1038/ncomms6437

**Published:** 2014-11-25

**Authors:** Omer Ziv, Amit Zeisel, Nataly Mirlas-Neisberg, Umakanta Swain, Reinat Nevo, Nir Ben-Chetrit, Maria Paola Martelli, Roberta Rossi, Stefan Schiesser, Christine E. Canman, Thomas Carell, Nicholas E. Geacintov, Brunangelo Falini, Eytan Domany, Zvi Livneh

**Affiliations:** 1 Department of Biological Chemistry, Weizmann Institute of Science, Rehovot 76100, Israel; 2 Department of Physics of Complex Systems, Weizmann Institute of Science, Rehovot 76100, Israel; 3 Department of Biological Regulation, Weizmann Institute of Science, Rehovot 76100, Israel; 4 Institute of Hematology, University of Perugia, Perugia 06100, Italy; 5 Department of Chemistry, Ludwig Maximilians University Munich, Munich 81377, Germany; 6 Department of Pharmacology, University of Michigan, Ann Arbor, Michigan 48109, USA; 7 Department of Chemistry, New York University, New York, New York 10003, USA

## Abstract

Cells cope with replication-blocking lesions via translesion DNA synthesis (TLS). TLS is carried out by low-fidelity DNA polymerases that replicate across lesions, thereby preventing genome instability at the cost of increased point mutations. Here we perform a two-stage siRNA-based functional screen for mammalian TLS genes and identify 17 validated TLS genes. One of the genes, *NPM1*, is frequently mutated in acute myeloid leukaemia (AML). We show that NPM1 (nucleophosmin) regulates TLS via interaction with the catalytic core of DNA polymerase-η (polη), and that NPM1 deficiency causes a TLS defect due to proteasomal degradation of polη. Moreover, the prevalent *NPM1c+* mutation that causes NPM1 mislocalization in ~30% of AML patients results in excessive degradation of polη. These results establish the role of NPM1 as a key TLS regulator, and suggest a mechanism for the better prognosis of AML patients carrying mutations in NPM1.

## Abstract (web summary)

Cells cope with replication-blocking DNA lesions by translesion DNA synthesis (TLS) polymerases, including polη. Here, the authors show that *NPM1*, a gene frequently mutated in acute myeloid leukaemia, protects polη from proteasomal degradation, and that NPM1 deficiency causes a TLS defect.

## Graphical abstract

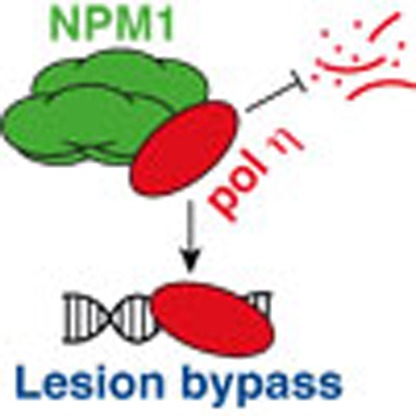

The high abundance of DNA damage has led to the evolution of a variety of DNA-damage tolerance and repair pathways that minimize potential deleterious effects on DNA replication and gene expression, thereby preventing genome instability and a plethora of pathological conditions^1^. Most of the damage is repaired by accurate DNA repair mechanisms, primarily during the G1 phase of the cell cycle. However, during S phase, DNA replication encounters DNA lesions that have escaped repair, or that were newly formed, leading to the arrest of replication forks and/or the formation of single-stranded gaps, which may further lead to the formation of double-stranded breaks (DSB) and genome instability^2^. These replication obstacles are dealt with by DNA-damage tolerance^3^, of which two main strategies are known: (1) translesion DNA synthesis (TLS), whereby specialized low-fidelity DNA polymerases replicate across the damaged DNA region in a process that is inherently error prone^4^,^5^,^6^,^7^; and (2) homology-dependent repair in which the gap opposite the DNA lesion is filled-in by either physical transfer of the complementary strand from the sister chromatid or by using the latter as a template for copying the missing strand (also termed error-free post-replication repair or template switch repair)^8^,^9^,^10^,^11^,^12^,^13^. The importance of TLS is highlighted by the hereditary disease xeroderma pigmentosum variant (XPV), which is characterized by sunlight sensitivity and very high predisposition to skin cancer, caused by germline mutations that inactivate DNA polymerase-η (polη), a major TLS DNA polymerase^14^,^15^. The realization that TLS maintains a low mutagenic burden despite its inherent error-prone nature, and protects cells against genome instability and cancer, raised great interest in this process^5^,^6^. TLS usually involves two DNA polymerases: an inserter, which incorporates a nucleotide opposite the damaged template base, and an extender, which continues DNA synthesis beyond the damaged base^16^,^17^. Several layers of TLS regulation are known, including damaged-induced monoubiquitination of proliferating cell nuclear antigen (PCNA), the sliding DNA clamp, which serves to recruit TLS DNA polymerases to damaged sites in the DNA^18^,^19^,^20^,^21^,^22^,^23^,^24^ and clearance of TLS polymerases from the DNA by the activity of DVC1 and p97 (refs 21, 22). In terms of cell physiology, TLS largely operates uncoupled from DNA replication, during late S and early G2 phases of the cell cycle^25^,^26^,^27^, and is also regulated by the DNA-damage response via the ataxia telangiectasia and Rad3-related (ATR) protein^28^,^29^ and via p53/p21 (refs 30, 31, 32). The high complexity of TLS in mammalian cells and its involvement in the development of cancer drug resistance^33^,^34^,^35^,^36^,^37^ highlights the importance of understanding how this process is regulated. While previous studies aimed to systematically identify TLS genes in *Saccharomyces cerevisiae* proved to be highly useful in the field, to the best of our knowledge, screening for mammalian TLS genes has not been yet reported, and high-throughput assays for mammalian TLS are currently not available.

Here we present the development of a high-throughput assay for TLS in mammalian cells, and its implementation in screening 1,000 candidate genes. We further describe the validation of 17 novel TLS players, and the mechanistic and clinical insights revealed by investigating one of them, nucleophosmin, encoded by the *NPM1* gene. We show that NPM1 regulates TLS by protecting polη from proteasomal degradation, and that a deficiency in NPM1 as well as expression of the acute myeloid leukaemia (AML)-related NPM1c+ mutation results in decreased polη levels and defective TLS. Our results uncover multiple novel TLS regulators in mammalian cells and implicate NPM1 in the proteolytic regulation of TLS polymerases.

## Results

### Two-stage functional siRNA screen for mammalian TLS genes

**Figure 1:**
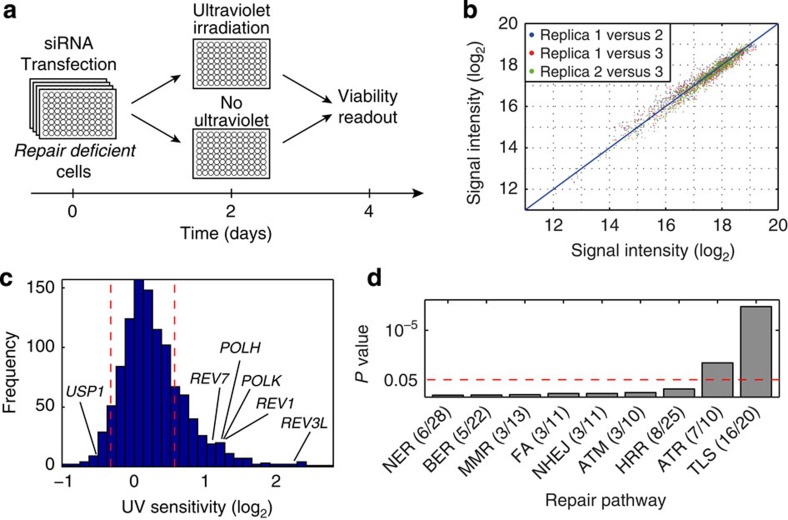
Primary screen for ultraviolet sensitivity in NER-deficient human cells. (**a**) An outline of the primary screen. In brief, NER-deficient *XPA* cells were transfected with the siRNA libraries on day 0, exposed to ultraviolet C (UVC) irradiation (1 J m^−2^) on day 2 and analysed for cell viability on day 4. (**b**) Data reproducibility. Luminescence values of three biological replicas that were preformed on different days were plotted against each other. (**c**) Histogram describing ultraviolet-sensitivity fold change (FC) caused by the siRNAs (median values over the three replicas). Dashed red lines denote the distribution borders of negative control samples. (**d**) Enrichment of TLS, DNA repair and DNA-damage response pathways within the screen hits. Dashed red line corresponds to a hypergeometric test *P* value 0.05. NER, nucleotide excision repair; BER, base excision repair; MMR, mismatch repair; FA, the Fanconi anemia pathway; NHEJ, non-homologous end joining; HRR, homologous recombination repair; ATM and ATR, the two main DNA-damage response pathways. See also Supplementary Fig. 1, Supplementary Data 1 and Supplementary Table 1.

We performed a two-stage functional short interfering RNA (siRNA) screen designed to identify new mammalian TLS genes. In the first stage, we assayed ultraviolet sensitivity using an *XPA* cell line that is deficient in nucleotide excision repair (NER), and therefore defective in the repair of ultraviolet-induced DNA damage. Consequently, ultraviolet survival of the *XPA* cells exhibits a greater dependence on DNA-damage tolerance compared with NER-proficient cells^38^, making the screen more selective to DNA-damage tolerance genes. siRNAs that were identified in this stage as significantly affecting ultraviolet survival were re-screened with a second more stringent assay, which measured TLS. This strategy was used to screen 1,000 siRNAs directed to genes involved in DNA repair, ubiquitination and de-ubiquitination, cell cycle regulation and cancer. The ultraviolet-sensitivity screen (Fig. 1a) was performed in three biological replicas, exhibiting good reproducibility (Fig. 1b). Of the 1,000 genes assayed, we found 192 genes for which knockdown resulted in elevated ultraviolet sensitivity, and 45 genes that reduced ultraviolet sensitivity (false discovery rate (FDR) <8%; Fig. 1c, Supplementary Data 1, Supplementary Table 1 and Supplementary Fig. 1). Known TLS genes, as well as genes related to the ATR DNA-damage response pathway, but not other DNA repair pathways, were highly represented among the hits (Fig. 1d), suggesting that the *XPA* deficiency indeed enriched for genes involved in TLS.

**Figure 2:**
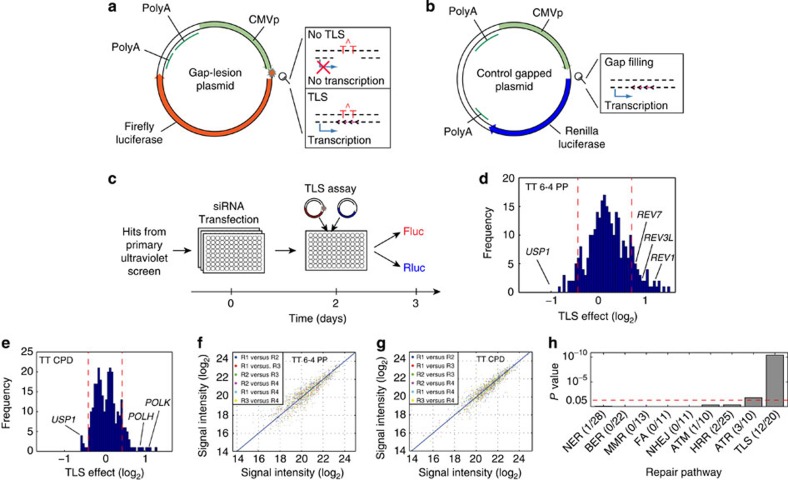
Secondary functional TLS screen. (**a**,**b**) The plasmids used for the secondary TLS screen: A *Fluc*-expressing gapped plasmid carrying a site-specific DNA lesion on the non-transcribed strand and a gap on the transcribed strand (**a**) and a control *Rluc*-expressing gapped plasmid without a lesion (**b**). (**c**) An outline of the secondary TLS screen. In brief, *XPA* cells were transfected with the siRNA on day 0 and with the reporter plasmids on day 2. Fluc and Rluc signals were measured on day 3. (**d**,**e**) Histograms describing TLS FC effects caused by the siRNAs (median values over four replicas that were preformed on different days). Dashed red lines denote the distribution borders of negative control samples. Data from the TT 6-4 PP TLS screen is presented in **d**, and that from the TT CPD TLS screen in **e**. (**f**,**g**) Luminescence values of the four biological replicas plotted against each other from the TT 6-4 PP screen (**f**), and the TT CPD screen (**g**). (**h**) Enrichment of TLS, DNA repair and DNA-damage response pathways within the screen hits. The dashed red line corresponds to a hypergeometric test *P* value 0.05. See also Supplementary Fig. 2, Supplementary Data 1 and Supplementary Tables 1 and 2.

The second screening stage was performed using a newly developed high-throughput TLS assay. It is a modification of an assay based on plasmids containing a site-specific DNA lesion opposite a gap, previously successfully used to study TLS^17^,^32^,^38^. In the new assay, the gap lesion is positioned between a cytomegalovirus (CMV) promoter and a firefly luciferase (*Fluc*) reporter gene (Fig. 2a). The presence of a gap in the transcribed strand does not allow expression of the *Fluc* gene, unless the missing segment of the coding strand is synthesized by TLS. A lesion-free gapped plasmid expressing *Renilla* luciferase (*Rluc*) served to normalize for transfection and gap-filling efficiencies (Fig. 2b). Of note, the product of a successful TLS event in the *Fluc* system still carries the lesion on the non-transcribed strand, which might interfere with *Fluc* expression. To test this possibility, we constructed the expected TLS product, namely a fully double-stranded plasmid with the lesion on the non-transcribed strand, and found that its *Fluc* expression in *XPA* cells was essentially identical to that obtained with a control plasmid without the lesion (Supplementary Fig. 2a).

The high-throughput TLS system was utilized to screen 237 siRNA hits from the primary screen for involvement in TLS across each of two major ultraviolet irradiation-induced DNA lesions, namely thymine–thymine 6-4 photoproduct (TT 6-4 PP) and thymine–thymine cyclobutane pyrimidine dimer (TT CPD; Fig. 2c–e). Screening was performed in four independent biological replicas performed on different days and exhibited high reproducibility (Fig. 2f,g). To normalize for differential damage-independent knockdown effects on *Fluc* and *Rluc* expression, each siRNA was also tested using *Fluc* and *Rluc* plasmids both carrying gaps but no lesions (NLs), and the results served to correct the data (Supplementary Fig. 2b,c). TLS elevation or inhibition scores were calculated with respect to control siRNAs, and were subjected to statistical analysis (Supplementary Fig. 2d–f). This screen resulted in 47 novel hits and 12 known TLS genes (FDR <10%; Supplementary Data 1 and Supplementary Table 1). Importantly, known TLS genes, but not other DNA repair pathways, were further enriched among the hits (Fig. 2h and Supplementary Table 2), indicating high specificity of the screening method towards the TLS pathway. The ATR signalling pathway was also enriched to some extent, in agreement with recent reports^28^,^29^. Repetitive examination of each hit using four different siRNA oligos, to minimize off-target effects, excluded 30 of the genes, yielding 17 validated novel TLS genes (Table 1).

### Functional validation of candidate TLS genes

**Figure 3:**
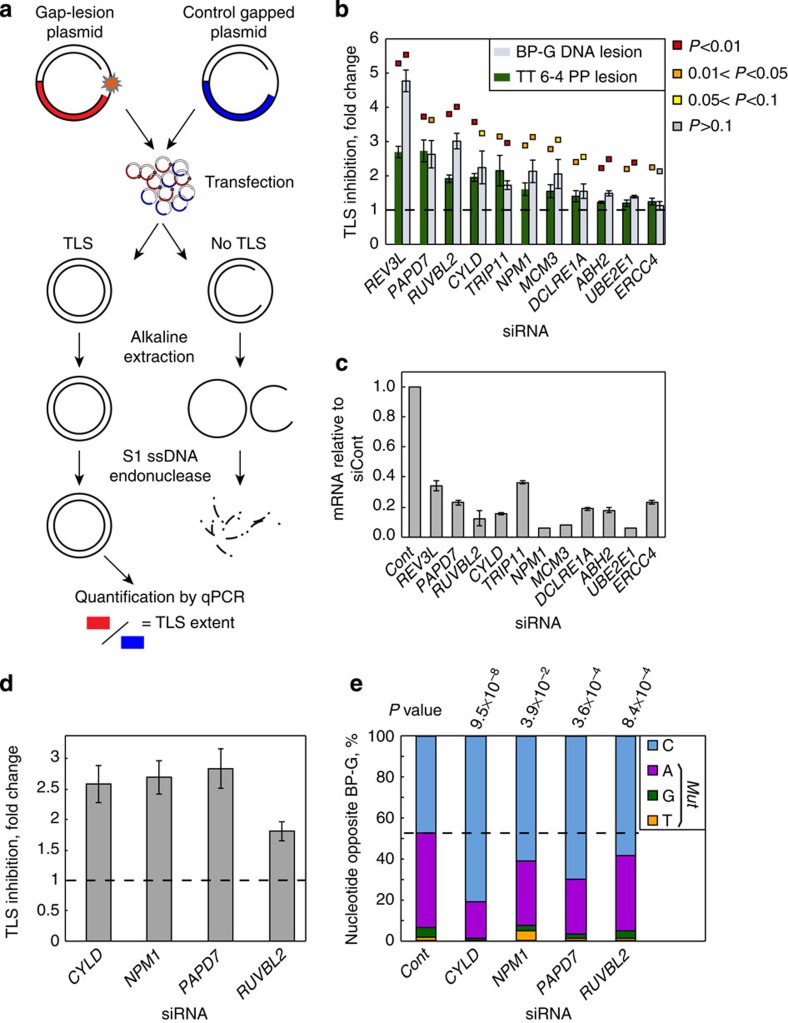
Validation of TLS candidate genes by qPCR-based and colony-based TLS assays. (**a**) An outline of the qPCR-based TLS assay. In brief, following TLS in mammalian cells, the plasmids were extracted under alkaline conditions, and subsequently treated with single-stranded-specific endonuclease. This ensures that only filled-in plasmids remain intact. TLS efficiency was calculated as the ratio between the products of qPCRs targeting the filled-in gap-lesion plasmid, and the filled-in control gapped plasmid. (**b**) qPCR-based TLS assays in MEFs. Ten hit genes were examined for involvement in TLS across the tobacco smoke-induced lesion BP-G and the ultraviolet-induced damage TT 6-4 PP, with Rev3L, encoding the catalytic subunit of DNA polymerase-ζ, serving as a positive control. Data are represented as mean±s.e.m. of 4–6 biological replicas. One-sample *t*-test, *P* values are indicated as a colour code above each column. (**c**) mRNA knockdown efficiencies by the siRNAs used in **b**. Values were measured by qPCR and normalized to sample treated with control siRNA (siCont). Mean values±s.e.m. of three replicas are presented. (**d**) Colony-based TLS assay across BP-G in mouse cells. Data are represented as mean±s.e.m. of three biological replicas. All the tested genes gave two-tailed *t*-test, *P* values <0.02. (**e**) Mutagenicity of TLS as observed in the colony-based TLS assays shown in **d**. Ninety-six colonies were analysed per condition. *χ*^2^ *P* values were calculated relative to control siRNA. ssDNA, single-stranded DNA.

To further validate the hits, a second version of the TLS assay was developed based on quantitative PCR (qPCR) readouts rather than gene expression (Fig. 3a). In brief, following TLS in mammalian cells, the plasmids were extracted under alkaline conditions, which denatured gapped plasmids but not the covalently closed products of TLS or the control gap-filling reactions. Remnants of gapped plasmids were digested with S1 single-stranded endonuclease. TLS efficiency was calculated as the ratio between the products of qPCRs that targeted the filled-in gap-lesion plasmid and the filled-in control gapped plasmid, and was normalized to control siRNA-treated samples (Fig. 3a). The qPCR-based TLS assay was utilized to examine the involvement of 10 selected hits in TLS across the TT 6-4 PP ultraviolet lesion in mouse embryonic fibroblasts (MEFs). In parallel, TLS was measured across the major tobacco smoke-induced DNA adduct benzo[*a*]pyrene-guanine (BP-G), a non-ultraviolet lesion. Six hits, namely *Papd7*, *Ruvbl2*, *Trip11, Npm1*, *Abh2* and *Ube2e1,* significantly affected TLS across both DNA lesions, although the effects obtained for *Abh2* and *Ube2e1* were rather small (Fig. 3b, *t*-test, *P* values <0.05; knockdown efficiencies are shown in Fig. 3c). *Cyld*, *Mcm3*, *Dclre1a* and *Ercc4* affected TLS across TT 6-4 PP but not across BP-G (Fig. 3b, *Cyld* knockdown reduced TLS across BP-G, but gave a marginal *P* value of 0.06). Of notice, all the 10 tested human genes were validated in the qPCR-based TLS assay with at least one DNA lesion in MEFs. Four genes were further tested for their impact on TLS extent and mutagenicity in a gapped plasmid assay in which the readout is based on transformation of *Escherichia coli* cells, and therefore enables determining the sequence signature of TLS events^17^,^32^,^38^. Knocking down the expression of each of the tested genes, namely *Cyld*, *Npm1*, *Papd7* and *Ruvbl2*, significantly reduced TLS efficiency across the BP-G adduct (Fig. 3d; two-tailed *t*-test, *P* values <0.02). DNA sequence analysis showed that in control cells TLS was 47% accurate (insertion of a dCMP opposite BP-G), and 53% were point mutations, most of which (87%) were caused by insertion of dAMP opposite the lesion (Fig. 3e), consistent with previous results^17^,^39^. Interestingly, knocking down the expression of each of the four tested genes also caused, in addition to the decrease in the extent of TLS, a lower error rate among the TLS events (Fig. 3e).

### NPM1 regulates DNA polη promoted TLS

**Figure 4:**
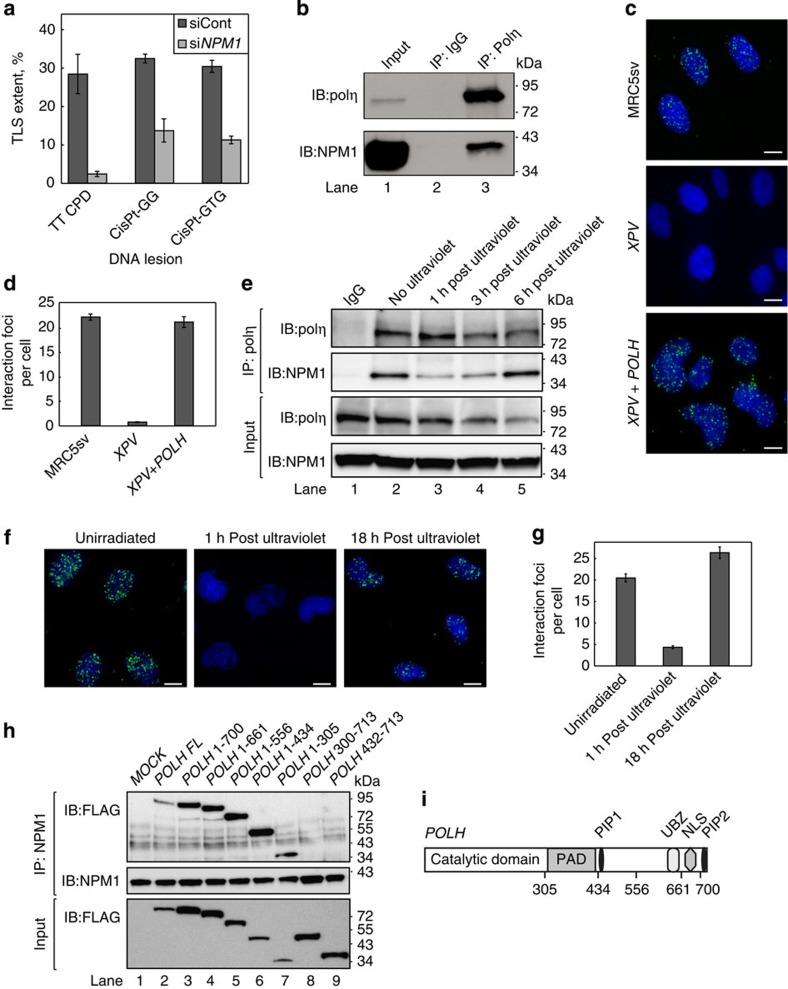
NPM1 interacts with DNA polη and regulates polη-promoted TLS. (**a**) NPM1 is required for effective TLS across site-specific TT CPD, or the cisplatin-induced site-specific lesions cisPt-GG, and cisPt-GTG in MEFs. Results of gapped plasmid-based, colony-forming TLS assays are presented as the mean±s.e.m. of three biological replicas. Two-tailed *t*-test, *P* values <0.02 for all tested lesions. (**b**) Co-IP of polη and NPM1 in extracts from MRC5sv cells. (**c**) PLA of polη and NPM1 in MRC5sv cells (upper panel), polη-deficient *XPV* cells (middle panel) and *XPV* cells complemented with ectopic polη expression (bottom panel). Blue, DAPI (4,6-diamidino-2-phenylindole) staining of the DNA in the nucleus; green, polη-NPM1 interaction detected by the assay. White scale bars correspond to 10 μm. (**d**) Quantification of the interacting foci shown in **c**, based on at least 140 cells of each type. Mean values±s.e.m. are presented. (**e**–**g**) Polη-NPM1 interaction is transiently lost following ultraviolet irradiation. (**e**) Co-IP of polη and NPM1 extracted from MRC5sv cells at different time points after ultraviolet irradiation with 10 J m^−2^. (**f**) PLA of polη and NPM1 in unirradiated and ultraviolet irradiated cells. Blue, DAPI staining of the DNA in the nucleus; Green, polη–NPM1 interaction. White scale bars correspond to 10 μm. (**g**) Quantification of the interacting foci shown in **f**, based on at least 100 cells of each type. Mean values±s.e.m. are presented. (**h**) NPM1 interacts with the catalytic core of polη. NPM1 was precipitated from HEK293FT cells expressing a serious of FLAG-POLH deletion mutants. Co-precipitation of the FLAG-POLH mutants is shown in the upper panel, whereas the expression of POLH mutants before precipitation is shown in the lower panel. (**i**) The functional domains of polη. PAD, polymerase-associated domain; PIP1 and PIP2, PCNA-interacting protein domains 1 and 2, respectively; UBZ, ubiquitin-binding zinc finger; NLS, nuclear localization signal. All blots are representative of three independent experiments. IB, immunoblotting; siCont, control siRNA. POLH FL, POLH full length.

Among the novel TLS genes, we concentrated for further analysis on *NPM1*, a gene frequently mutated in AML^40^. NPM1 (also termed nucleophosmin or B23) is a multi-functional protein involved in diverse cellular processes such as ribosome biogenesis, histone assembly, protein chaperoning and cell proliferation^41^. To explore the possibility that NPM1 affects polη-promoted TLS, we tested TLS across three lesions that are bypassed by polη: the sunlight-induced TT CPD and two adducts formed by the chemotherapy drug cisplatin, namely cisPt-GG and cisPt-GTG. As can be seen in Fig. 4a, in MEF cells in which *Npm1* was knocked down, TLS across each of the three lesions was significantly reduced (two-tailed *t*-test, *P* values <0.02), with the TT CPD being most affected. In contrast, knocking down *Npm1* in polη-deficient *PolH*^*−/−*^ MEF cells did not significantly affect TLS across the TT CPD lesion (Supplementary Fig. 3a,b), further suggesting that the effect of NPM1 on TLS across TT CPD is mediated via polη. This effect was not indirectly mediated via APE1, the base excision repair endonuclease that is regulated by NPM1, since knocking down the expression of *Ape1* in the MEF cells had no effect on TLS across TT CPD (Supplementary Fig. 3c,d). Since NPM1 was reported to control the levels of PCNA^42^, we examined whether the effect on TLS is via reducing PCNA monoubiquitination, which is needed for effective recruitment of polη to damaged sites in DNA^19^,^20^ and for effective TLS^23^. Interestingly knocking down the expression of *NPM1* increased PCNA monoubiquitination rather than decreasing it (Supplementary Fig. 4). This rules out the possibility that the TLS deficiency is owing to a PCNA-ubiquitination defect. A possible explanation for this observation is that the TLS defect caused by knockdown of *NPM1* increases the number of stalled replication forks that leads to increased PCNA monoubiquitination.

### NPM1 interacts with DNA polη in the nucleus

We next examined the interaction between polη and NPM1 by co-immunoprecipitation (co-IP) of the endogenous NPM1 with endogenous polη. As can be seen in Fig. 4b, immunoprecipitation (IP) of polη with a specific antibody caused co-precipitation of NPM1. No NPM1 was observed when a control IgG was used (Fig. 4b, lane 2). To probe the subcellular compartment in which NPM1 and polη interact, we resorted to the proximity ligation assay (PLA). As can be seen in Fig. 4c, interaction foci between polη and NPM1 were clearly observed in human MRC5sv cells and localized mainly in the nucleus (the green spots in Fig. 4c upper panel, and quantified in Fig. 4d). These interaction foci were not observed in control *XPV* cells that lack polη (Fig. 4c middle panel, and Fig. 4d), but appeared in *XPV* cells complemented with the *POLH* gene, encoding polη (Fig. 4c lower panel, and Fig. 4d). The interaction foci appear to be distributed throughout the nucleus but were excluded from the nucleoli (Supplementary Fig. 5a). Similarly, in MRC5sv cells co-expressing green fluorescent protein (GFP)-tagged polη and mCherry-tagged NPM1, GFP-polη was distributed throughout the nucleus and in reduced amounts in the nucleoli (Supplementary Fig. 5b). Taken together, these results indicate that NPM1 interact with polη in the nucleoplasm.

We next examined whether DNA-damage induction affects the interaction between NPM1 and polη. Exposure of the MRC5sv cells to ultraviolet irradiation resulted in a decrease in the amount of polη while the total levels of NPM1 were not significantly altered (Fig. 4e, input blot). Interestingly, following ultraviolet irradiation the interaction between polη and NPM1 was transiently lost, and reappeared at later time points, as detected by co-IP (Fig. 4e). Similarly, analysis by the PLA showed a transient loss of interaction between polη and NPM1 following ultraviolet irradiation (Fig. 4f, quantified in Fig. 4g). Ultraviolet irradiation of MRC5sv cells co-expressing GFP-tagged polη and mCherry-tagged NPM1 resulted in recruitment of polη but not of NPM1 into replication foci (Supplementary Fig. 5b), further supporting the observation that NPM1 releases polη following ultraviolet irradiation. To identify the region of polη that interacts with NPM1, a series of FLAG-polη deletion mutants was constructed and expressed in HEK293 cells. While IP of NPM1 resulted in co-precipitation of FLAG-polη mutants lacking its regulatory carboxy terminus (Fig. 4h lanes 2–7, and Fig. 4i), it failed to co-precipitate the FLAG-polη mutants lacking the catalytic core of the polymerase (Fig. 4h lanes 8-9, and Fig. 4i). This suggests that NPM1 interacts with the catalytic core of polη.

### NPM1 protects DNA polη against proteasomal degradation

**Figure 5:**
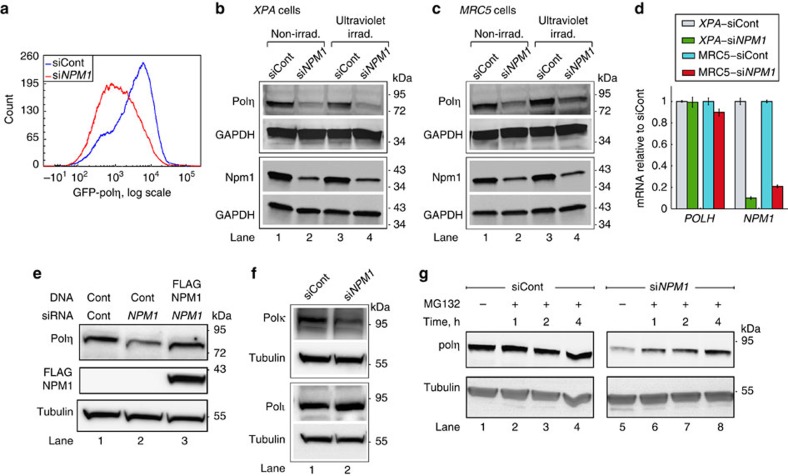
NPM1 protects DNA polη from proteasomal degradation. (**a**) MRC5sv cells stably expressing *GFP-POLH* were treated with siRNA against *NPM1* or non- targeting control siRNA (siCont). GFP-polη levels were quantified by flow cytometry. (**b**,**c**) Immunoblot of polη extracted from human *XPA* cells (**b**) and MRC5sv cells (**c**) pretreated with siRNA against *NPM1* or non-targeting siCont. When indicated, cells were ultraviolet-irradiated 15 h before extraction with 2 J m^−2^ (**b**) or 10 J m^−2^ (**c**). Upper and lower blots were done with the same extracts. (**d**) Polη downregulation is not at the transcription level. qPCR of *POLH* and *NPM1* mRNA levels from cells pretreated with siRNA against *NPM1*, normalized to non-targeting siRNA-treated cells. Mean values±s.e.m. of three replicas are presented. (**e**) Immunoblot of polη extracted from MRC5sv cells pretreated with siRNA against *NPM1* and complemented by ectopic expression of an siRNA-resistant *NPM1* construct. (**f**) Polκ, but not polι, is also affected by *NPM1* knockdown. Immunoblots of polκ and polι extracted from MRC5sv cells pretreated with siRNA against *NPM1*, or non-targeting siCont. (**g**) Accumulation of polη following proteasomal inhibition. MRC5sv cells were treated with non-targeting siRNA or siRNA against *NPM1* and then subjected to MG132 for the indicated time periods in order to inhibit proteasomal activity. Tubulin served as a loading control. All blots are representative of three independent experiments. Irad., irradiated.

To examine whether NPM1 affects polη stability, we measured by flow cytometry the level of ectopically expressed *GFP-POLH* in MRC5sv cells that stably express this construct. Knocking down *NPM1* in these cells resulted in a threefold decrease in the expression of the GFP-polη construct (Fig. 5a, median values). When cells expressing *GFP*-*RAD18*, a gene fusion of the main E3 ligase responsible for monoubiquitination of PCNA were analysed, no reduction in RAD18 expression was obtained following knockdown of *NPM1* (Supplementary Fig. 6), which is in agreement with the functional PCNA ubiquitination in these cells. Next, we tested whether NPM1 regulates the protein level of endogenous polη. Knocking down the expression of *NPM1* in *XPA* cells caused a significant fourfold decrease in the amount of the NPM1 protein (Fig. 5b, lanes 1 and 2). A similar result was obtained with the MRC5sv cells (3.4-fold decrease; Fig. 5c, lanes 1 and 2). Under these conditions, the amount of polη was significantly reduced both in the *XPA* cells (3.8-fold decrease; Fig. 5b, lanes 1 and 2) and the repair-proficient MRC5sv cells (2.9-fold decrease, Fig. 5c, lanes 1 and 2), with no significant change in *POLH* mRNA level, as measured by qPCR (Fig. 5d). A similar effect was observed in ultraviolet-irradiated cells (Fig. 5b lanes 3 and 4, and Fig. 5c lanes 3 and 4, corresponding to *XPA* and MRC5sv cells, respectively). Importantly, ectopic expression of an siRNA-resistant *NPM1* construct rescued the reduction in polη caused by the silencing of endogenous NPM1 (Fig. 5e). Thus, a reduction in the amount of NPM1 causes a decrease in the amount of endogenous polη at the post-transcriptional level. A similar reduction was also observed with polκ, which is another TLS DNA polymerase, but not with polι, the closest homologue of polη in mammalian cells (Fig. 5f). Inhibiting the proteasome using MG132 caused an increase in polη levels in cells pre-treated with siRNA against *NPM1*, but not in those treated with control siRNA (Fig. 5g). This indicates that NPM1 has an important function in maintaining the stability of polη, and protecting it from proteasomal degradation. The E3 ubiquitin ligases PIRH2 and MDM2 were implicated in the proteasomal degradation of polη. Nevertheless, knocking down each of these enzymes or both, in addition to knocking down NPM1, did not rescue the polη level (Supplementary Fig. 7a,b), suggesting that the degradation of polη upon NPM1 deficiency is not mediated solely by PIRH2 or by MDM2.

### *NPM1c+* mutations phenocopy the deficiency in DNA polη

**Figure 6:**
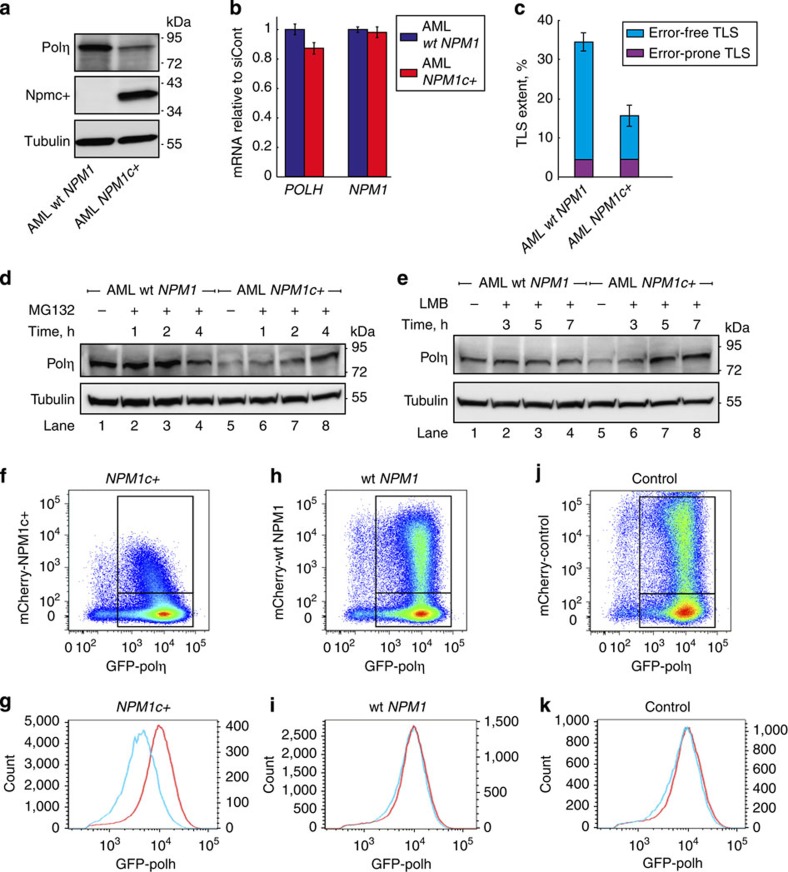
The NPM1c+ mutation causes DNA polη degradation and TLS deficiency. (**a**) Immunoblot of polη extracted from OCI/AML2 and OCI/AML3 cells expressing wt *NPM1* and *NPM1c+* alleles, respectively. (**b**) qPCR of *POLH* and *NPM1* mRNA levels from OCI/AML2 and OCI/AML3 cells. Mean values±s.e.m. of three replicas are presented. (**c**) TLS in OCI/AML2 and OCI/AML3 cells. TLS across a site-specific TT CPD using the gapped plasmid-based, colony-forming TLS assay is shown. Mean values±s.e.m. of three replicas are presented, two-tailed *t*-test, *P* value <0.003). Mutagenicity was addressed by sequencing 96 TLS events per cell line, *χ*^2^-test, *P* value <0.01. (**d**) Proteasomal inhibition rescued Polη deficiency in *NPM1c+* AML cells. Polη levels in OCI/AML2 and OCI/AML3 cells pretreated with MG132 for the indicated time to inhibit proteasomal activity. Tubulin served as a loading control. (**e**) NPM1 nuclear retention rescued Polη deficiency in *NPM1c+* AML cells. Polη levels in OCI/AML2 and OCI/AML3 cells pretreated with Leptomycin B (LMB) for the indicated time or its solvent as a control. Tubulin served as a loading control. (**f**–**k**) Density plots and histograms representing fluorescence-activated cell sorting analysis of HEK293 cells stably expressing GFP-polη, and partially transfected with mCherry-tagged NPMc+ (**f**,**g**), wt NPM1 (**h**,**i**) and control mCherry (**j**,**k**). Data from mCherry-negative (untransfected) cells in (**g**,**i**,**k**) are in red and correspond to the left *y* axis, while that from mCherry-positive (transfected) cells are in blue and correspond to the right *y* axis. All blots are representative of three independent experiments. siCont, control siRNA.

The C terminus of NPM1 is mutated in ~30% of all AML patients, resulting in loss of the nucleolar localization signal and the generation of a *de novo* nuclear export signal. This leads to mislocalization of mutant NPM1 (designated NPM1c+), and partially of wild-type (wt) NPM1 interacting with it, in the cytoplasm^40^,^43^. These mutations strongly correlate with better response of patients to chemotherapy and better clinical outcome^44^,^45^. Because of the role of NPM1 in stabilizing polη in the nucleus, we hypothesized that mislocalization of NPM1 to the cytoplasm might cause destabilization of polη, similar to the effect that we have observed in cells in which NPM1 was knocked down. To this end, we examined polη levels in OCI/AML2 and OCI/AML3 cell lines derived from AML patients, either without or with the *NPM1c+* mutation, respectively. As can be seen in Fig. 6a, the amount of polη was much lower in the *NPM1c+* AML cell line compared with the *NPM1*wt AML cell line, whereas the *POLH* mRNA levels were similar (Fig. 6b). Consistently with the lower polη amount, TLS across a TT CPD was lower (two-tailed *t*-test, *P* value <0.003), and more mutagenic (*χ*^2^-test, *P* value <0.01) in the *NPM1c+* AML cells compared with the *NPM1*wt AML cells (Fig. 6c). Proteasome inhibition using MG132 rescued the level of polη in the *NPM1c+* AML cell line (Fig. 6d), indicating that polη is subjected to excessive proteasomal degradation, as was the case in cells in which *NPM1* was knocked down. To further support the notion that it is the mislocalization of NPM1 in the AML cells that causes the polη deficiency, we treated the cells with Leptomycin B, an inhibitor of nuclear export via exportin 1, previously shown to prevent the exclusion of NPM1 from the nucleus^46^. As can be seen in Supplementary Fig. 8, NPM1 was present mainly in the cytoplasm in *NPM1c+* AML cells, but not in the *NPM1*wt AML cells, as expected. Upon addition of Leptomycin B, The *NPM1c+* AML cells exhibited nuclear localization of NPM1, similarly to the *NPM1*wt cells. Examining the level of polη under these conditions revealed that Leptomycin B treatment did not change the amount of polη in *NPM1*wt AML cells, but caused a significant increase in polη in the *NPM1c+* AML cells (Fig. 6e), consistent with a role of NPM1 in stabilizing polη in the nucleus. Furthermore, ectopic expression of mCherry-NPM1c+ in HEK293 cells that stably express GFP-polη caused a significant twofold decrease in the amount of GFP-polη as indicated by flow cytometry analysis (Fig. 6f,g). This effect was not observed when mCherry-NPM1wt or mCherry alone were expressed (Fig. 6h–k). Taken together, these results establish a role for NPM1 in the regulation of TLS, and suggest that the prevalent NPM1c+ mutation results in reduced levels of polη, thereby leading to defective TLS.

## Discussion

The multiplicity of TLS DNA polymerases and their low fidelity suggest the existence of a complex regulation to ensure their action at the right place and the right time. The molecular basis of the regulation that enables a low and tolerable mutation burden is only partially understood. We reasoned that given its complexity, TLS in mammalian cells is regulated by additional as yet unidentified genes, and searched for those using a two-stage functional siRNA screen. Having two functional assays, one of which was specific for TLS, and a subsequent stringent validation protocol that included analysis by additional TLS assays, was anticipated to reduce the number of false hits. The high selectivity of the screens for TLS genes is demonstrated by the high enrichment of known TLS genes within the hits, in contrast to other DNA repair pathways. The switch to mouse cells for the subsequent qPCR validation enabled us to gain more validation power by using different siRNA oligos to target the mouse genes, and by demonstrating that the hits are not cell type- or human-specific. It should be noted that the secondary gapped plasmid-based screen used in this study measures TLS outside the chromosomal context, and hence TLS genes that are associated with chromatin structure or replication fork progression are unlikely to be scored. Such TLS genes might have been detected in the primary ultraviolet-sensitivity screen. The E3 ligase RAD18 that mediates PCNA monoubiquitination was not scored in our screen, although we have previously shown that it is required for effective TLS in the gap-lesion plasmid assay^23^. This might have been caused by inefficient knockdown of the gene. Our screening strategy had led to the identification of 17 new TLS genes, six of which, namely *NPM1*, *SMURF2*, *UBE2E1*, *CYLD*, *OTUB2* and *VCPIP1*, do not have obvious homologues in *E. coli* or *S. cerevisiae*. Of the 17 TLS genes, 10 were further examined by a transcription-/translation-independent qPCR version of the TLS assay in mouse cells, and at least four, namely *CYLD, NPM1, PAPD7* and *RUVBL2*, also affected mutation rates across the BP-G lesion, further supporting their role in TLS. Most of our hits were detected when screening for TLS across the TT 6-4 PP lesion, with only few exceptions detected in the TT CPD screen, perhaps owing to the robustness of CPD bypass by the highly specialized polη. Virtually, all of our hits seem to have a positive role in TLS, with the exception of USP1, which is a known negative TLS regulator responsible for de-ubiquitination of monoubiquitinated PCNA^47^.

Most of the new TLS hits can be classified into four functional categories, including DNA replication and repair genes, genes affecting proteasomal degradation, chaperons and Golgi-related genes (Table 1). The replication/repair category contains five genes, including the helicase subunit *MCM3*, which is involved in the formation of replication forks; *ALKBH2,* which is involved in a direct reversal repair pathway; *DCLRE1A*, which functions in the repair of DNA crosslinks; *ERCC4,* which function in HRR as well as in NER; and *RUVBL2*. Scoring those genes as regulators of TLS suggests cross talk between DNA replication, repair and damage tolerance. Interestingly, the recruitment of DCLRE1A to sites of damage requires the ubiquitination of PCNA by RAD18 in a similar manner to TLS polymerases^48^. Since TLS polymerases are implicated in crosslink repair, it is tempting to speculate that DCLRE1A participates in coordinating the TLS and crosslink repair pathways, a task in which FAAP20 was recently implicated^49^. RUVBL2 plays an essential part in several chromatin-remodeling complexes including INO80 that regulates transcription by sliding nucleosomes on DNA. Of note, the INO80 complex is also required for removal of histones at sites of damage and was recently suggested to regulate DNA-damage tolerance^50^,^51^.

The next two categories include four genes involved in protein degradation by the proteasome and two genes encoding chaperons, consistent with the importance of regulating the stability of TLS components. *UBE2E1* and *UBE2G2* are E2 ubiquitin ligases; *SMURF2* is an E3 ubiquitin ligase; and *RPN1* forms part of the regulatory subunit of the 26S proteasome. The chaperons group includes *NPM1,* which is discussed below, and *DNAJC6,* which belongs to the *DNAJ/HSP40* family of chaperons, and promotes uncoating of clathrin-coated vesicles. Of note, the *Caenorhabditis elegans* homologue of *DNAJC6* (*w07a8.3*) was previously scored as a hit in a mutagenesis screen, which further supports its involvement in TLS^52^. The category of Golgi-related genes includes *TRIP11* and *VCPIP1*. *VCPIP1* is a deubiquitinating enzyme that mediates the reassembly of Golgi stacks after mitosis. Importantly, it also interacts with *VCP/p97*, which was recently found to regulate TLS^21^,^22^,^53^. There are four additional TLS hits including two de-ubiquitination enzymes, namely *OTUB2* and *CYLD*, the latter being a negative regulator of the NF-κB pathway; *SENP2* that participates in SUMO signalling; and *PAPD7* that is an RNA-specific ribonucleotidyl transferase that plays a role in the turnover of aberrant mRNAs^54^. The new TLS genes, which belong to diverse pathways and functions, are likely to be useful for further analysis of TLS in mammalian cells and its interaction with other cellular pathways.

The finding that NPM1 is a regulator of TLS adds an unexpected dimension to the role of this multi-functional protein in preventing genome instability^55^,^56^,^57^, and uncovers a new key player of TLS regulation via proteasomal degradation. By protecting polη from degradation, NPM1 enables TLS to fulfil its function of overcoming replication obstacles, thereby preventing DSB and genome instability^58^. It was previously shown that two E3 ubiquitin ligases promote polyubiquitination of polη and target it for proteasomal degradation: *MDM2* (ref. 59) and *CRL4(CDT2)* (ref. 60). In addition, *PIRH2* targets polη to proteasomal degradation via a ubiquitination-independent pathway^61^. Thus, the cellular levels of polη are tightly regulated. Human *HSP90* was reported to facilitate the corrected folding of polη into its active form^62^, and *GEI-17* was shown to prevent the polyubiquitination and subsequent degradation of polη by *CRL4(CDT2)* following genotoxic stress in *C. elegans*^60^. While *GEI-17* is dispensable for polη stability at steady state, NPM1 is critical for maintaining a functional pool of polη in the absence of external DNA damaging agents. Thus, even if GEI-17 activity is conserved in mammals, it does not seem to overlap with the role of NPM1 in protecting polη stability, but rather work subsequently. The transient release of polη from NPM1 following ultraviolet irradiation might enable polη to reach damaged sites in the DNA through its affinity to monoubiquitinated PCNA and preform TLS, however, this dynamics of polη needs further investigation.

It was recently reported that polη is involved in the resistance to araC, a drug routinely used to treat AML, which exerts its therapeutic activity via incorporation into DNA and inhibiting replication^63^. Thus, our results may explain, at least in part, the better prognosis and response to chemotherapy treatment of AML patients carrying the NPM1c+ mutation, which causes exclusion of NPM1 from the nucleus, leading to a polη deficiency, and therefore more effective killing of the leukaemic cells by drugs such as araC. Polη was recently implicated in the cellular response to doxorubicin treatment^64^. Although we demonstrated that the NPM1c+ OCI/AML3 AML cells carry low levels of polη, they were reported to be more resistant to doxorubicin than OCI/AML2 cells^65^. The sensitivity of the AML cells to doxorubicin is most likely governed by processes other than TLS such as DSB repair and apoptosis, which might also be regulated by NPM1. Such processes can mask the effect of polη, which might be secondary for this drug. The results presented here underscore the critical role of NPM1 in maintaining genome stability, and highlight polη and NPM1, and in particular their interaction, as potential targets for development of drugs for AML therapy.

## Methods

### Plasmids construction

pGL4.13 and pGL4.73 reporter plasmids (Promega) served as backbones for the reporter gapped plasmids used in the screen. Restriction-free cloning was performed using PfuUltra II Fusion High-Fidelity DNA Polymerase (Stratagene). The following cloning steps were performed: (i) Bsa1 and BstX1 restriction sites were mutated; (ii) the SV40 promoter was replaced with a CMV promoter, taken from pRL-CMV (Promega); (iii) the ColE1 origin of replication was relocated to the region in between the CMV promoter and the reporter gene; and (iv) the AMP resistance cassette was replaced with a chloramphenicol resistance one, and was relocated in the firefly construct to the region in between the CMV promoter and the reporter gene. The mCherry-tagged NPM1 and NPM1c+ constructs were produced by fusing the mCherry coding region (Clontech) to the carboxyl terminus of the human *NPM1* or *NPM1c+* coding region. FLAG-tagged polη truncations were produced by introducing the FLAG epitope in the amino terminus of the human *POLH* coding region, and deleting the indicated fragments by restriction-free cloning. siRNA-resistant human *NPM1* construct was design by introducing silence mutations in the sequence corresponding to the siRNA oligo: 5′-GAGCACCAGUUAUCUUUAA-3′.

### Cell cultures

SV40-transformed MRC5sv, *XPA* (XP12RO), *XPV* (XP30RO) and *XPV*^*POLH+*^ human fibroblasts were gifts from A.R. Lehmann (University of Sussex, Brighton, UK). C57BL/6 MEFs were gifts from N. de Wind (Ludwig Maximilians University, Munich, Germany). OCI/AML2, OCI/AML3 and HEK293FT cells are commercially available. GFP-polη expressing cells were produced by infection of MRC5sv or HEK293FT cells with *GFP*-*POLH* encoding lentiviruses, followed by fluorescence-activated cell sorting (BD FACSAria) for GFP-expressing cells. Human fibroblasts were cultured in MEM, AML lines in MEM-α modification, and MEFs and HEK293FT in DMEM (Gibco). Each medium was supplemented with 2 mM alanyl-glutamine (Biological Industries), 100 units ml^−1^ of penicillin, 100 μg ml^−1^ of streptomycin (Biological Industries) and 10–15% fetal bovine serum (Gibco). Throughout the screening steps, cells were maintained at 37 °C in a 5% CO_2_ and 4% O_2_ atmosphere.

### Ultraviolet-sensitivity screen

Human *XPA* cells were reverse-transfected in 96-well plates with 25 nM siGenome SMARTpool siRNA libraries (Dharmacon), using Hiperfect transfection reagent (Qiagen). Following incubation of 2 days, cells were washed twice with Hanks’ buffer (Sigma) and irradiated in Hanks’ buffer at a dose of 1 J m^−2^ ultraviolet C using a low-pressure mercury lamp (TUV 15W G15T8, Philips). Following irradiation, the cells were maintained under growth conditions for 48 h. Cell viability was determined using CellTiter-Glo (Promega). In order to reduce misinterpretation of toxic effects of certain siRNAs, each sample was normalized using its corresponding non-irradiated sample transfected with the same siRNA. Ultraviolet sensitivity was calculated with respect to control wells.

### TLS screen

Human *XPA* cells were transfected with the siRNA hits from the ultraviolet-sensitivity screen at a concentration of 10 nM. The cells were incubated for 2 days, after which they were co-transfected with a mixture of 2.5 ng *Fluc* gap-lesion plasmid and 2.5 ng *Rluc* control gapped plasmid. Following incubation of 24 h, Fluc and Rluc signals were sequentially measured using the dual-luciferase reporter assay (Promega). TLS extent values were calculated as the ratio between Fluc and Rluc signals in respect to samples transfected with control siRNA, and were corrected for having two different reporters as described in the statistical analysis section. Exclusion of false-positive hits due to off-target effects of the siRNA was achieved by de-convolution of selected siRNA pools and testing each of the four siRNA oligos separately. Gap-lesion plasmids were constructed as described^66^.

### Statistical analysis of the primary TLS-specific screen

Viability was measured using luminescence readouts for a set of 1,100 siRNA pools with or without ultraviolet irradiation in three independent biological replicas. This can be represented by: *I*_UV*,ij*_; *I*_no UV*,ij*_ where ‘*i*’ stands for the gene index (siRNA pool) and ‘*j’* for the repeat index. All intensity values are given after log_2_ transformation. The assay has a 96-well format such that each plate contains nine negative controls of several kinds (non-targeting siRNA, RNA-induced silencing complex (RISC)-free siRNA and siRNA targeting *XPA*, which is already defective in the cells). Normalization was done by setting the mean intensity of the negative controls in each plate to be equal to the mean intensity of the negative control wells over all the plates. This was done separately for unirradiated plates and ultraviolet-irradiated plates, and the data were corrected accordingly. For each plate, the corresponding ultraviolet effect of the negative control wells is given by:


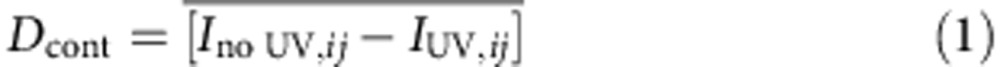


∀*_j_*=1, 2, 3, where average is over *i* ε negative control wells. Estimation of the noise level was done using intensity-dependent noise model^67^. This estimation assigns a s.d. value to each siRNA according to the mean intensity value. The approach was used since it provides better statistical power and is justified by the log Gaussian nature of the light intensity measurements. The statistic used for the analysis is:


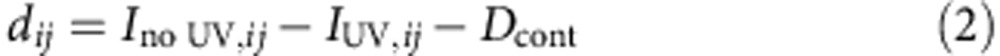


This represents the ultraviolet effect associated with a specific siRNA in a specific repeat with respect to the control ultraviolet effect. According to the null hypothesis, this statistic is distributed with zero mean and an intensity-dependent variance,


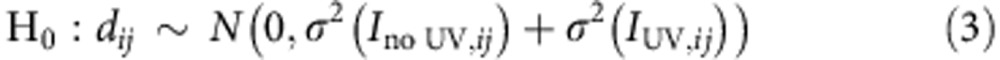


A *P* value was calculated based on this null hypothesis for each siRNA in each repeat. The three *P* values calculated for each siRNA were then combined using a Fisher meta *P* value to get a single *P* value per siRNA pool. We call a gene ‘ultraviolet sensitive’ if two conditions are satisfied: 8% FDR, and a median fold change (FC) of either FC >1.5 or FC <0.8; these values, which are represented by the red dashed vertical lines on Fig. 1b, were set according to the distribution of the negative control siRNAs.

### Statistical analysis of the secondary TLS-specific screen

The TT 6-4 PP screen, TT CPD screen and the NL correction assay where measured each in four independent biological repeats. Thus for each lesion, each siRNA (*i*) in each replica (*j*), we have the measured values: 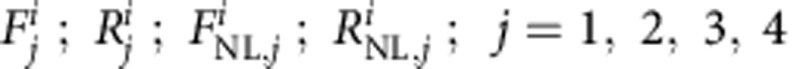, where ‘*F*’ stands for Fluc intensity and ‘*R*’ for Rluc intensity. Each measured value is compared with a similar experiment with the negative control siRNAs discussed above.

A natural statistic then is (the bar represents average over the four replicas):


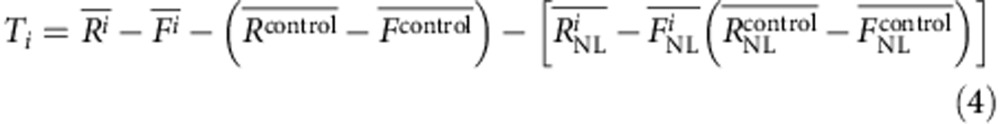


A difficulty arises when analysing this data, namely, the two numbers 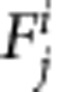 and 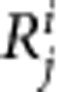 , measured from the same well, are strongly dependent in terms of transfection efficiency, which is not uniform between repeats and between wells in the same plate. The way we overcome this problem is by a normalization that keeps the difference in each well as was measured originally. This was done by setting the Rluc values to be the same for all replicates and the Fluc values to fit the differences.


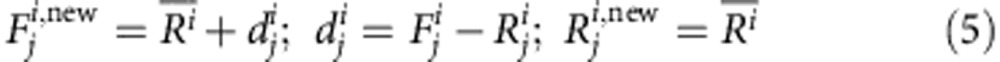


Under this normalization:


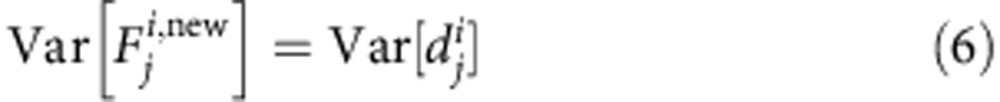


and the intensity distribution does not change. All three assays (TT 6-4 PP screen, TT CPD screen and NL correction) were normalized by this method. When using the new values, the variance of the statistic *T*_*i*_ defined above becomes:


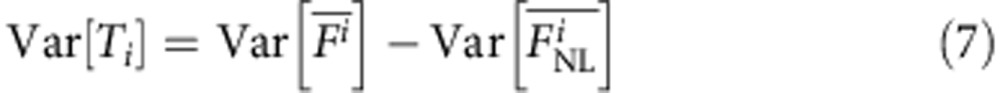


where the values 
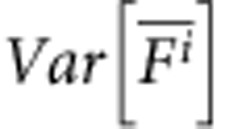
 are taken from intensity-dependent noise estimation, as was done for the ultraviolet-sensitivity screen. Thus, formulating the null hypothesis as:


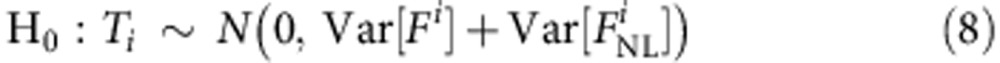


We chose a threshold that combines 10% FDR and a median FC of either FC >1.5 or FC <0.7 (represented by the red dashed vertical lines on Fig. 1f,g), which was set according to the distribution of the negative control siRNAs.

### qPCR-based and colony-based TLS assays

MEFs of wt origin were reverse-transfected with 10 nM siRNA. Following incubation for 2 days, the cells were co-transfected with the gap-lesion plasmid and the control gapped plasmid that had been used in the TLS screen. Cells were maintained for 12 h, after which their plasmid content was extracted under alkaline conditions, in which only covalently closed plasmids remained non-denatured. In the qPCR-based TLS assay, remnants of non-filled gapped plasmids were digested by the single-stranded DNA-specific S1-endonuclease (1.6 units μl^−1^, 30 min incubation). Reactions were terminated by adding 33 mM EDTA, followed by heat inactivation and purification by a Wizard SV PCR clean-up kit (Promega). TLS analysis was performed by qPCRs using a SYBR Green reagent (KAPA Biosystems). Discrimination between the filled-in gap-lesion plasmid and the control gapped plasmid was achieved based on two primer sets; one targeted the *Fluc* gene and the other the *Rluc* gene. The ratio between the two qPCR products, normalized to samples treated with control siRNA, served to assess the extent of TLS. In the colony-based TLS assay, the extracted plasmids were transformed into a TLS-defective *E. coli recA* strain, which was plated in parallel on Luria broth-kanamycin (for repaired gap-lesion plasmids) and Luria broth-chloramphenicol (for repaired control plasmids). The ratio of kanamycin/chloramphenicol resistant *E*. coli colonies represents the efficiency of TLS in the mammalian cells, and sequence analysis of plasmids extracted from individual colonies provides DNA sequence alterations during TLS. The siRNA sequences are listed in Supplementary Table 3.

### qPCR for mRNA evaluation

Total RNA was extracted from the cells 48–72 h after transfection with siRNA, using the Perfect-Pure RNA cultured cells kit (5-PRIME). cDNA was produced using High capacity cDNA reverse transcription kit (Applied Biosystems). A quantity of 5 ng cDNA was taken for qPCR analysis using SYBR Green reagent (KAPA Biosystems). The data was normalized according to measurements of the mouse *HPRT* and *RLP19* genes, or the human *GAPDH* and *HPRT* genes. The primers used for the qPCRs are listed in Supplementary Table 4.

### Immunoblot analysis

When indicated, cells were transfected with 15 mM siRNA (Dharmacon) using Hiperfect transfection reagent (Qiagen). Cells were maintained under growth conditions for 3 days, after which they were lysed using RIPA buffer supplemented with protease inhibitor cocktail and phenylmethanesulfonyl fluoride (PMSF) (Sigma). For PCNA-ubiquitination measurements, Triton-soluble fractions were extracted by incubating the cells for 5 min at 4 °C in buffer A (100 mM NaCl, 300 mM sucrose, 3 mM MgCl_2_, 10 mM PIPES (pH 6.8), 1 mM EGTA and 0.2% Triton X-100). Following removal of the buffer (Triton-soluble fraction), cells were rinsed once with cold PBS, and the adhering cellular material (Triton-insoluble fraction) was collected by scrapping and was extracted by incubating at 37 °C for 45 min in buffer B (same as buffer A but containing 50 mM NaCl and no Triton X-100) supplemented with 60 units DNaseI (Sigma). Protein concentration was determined using the BCA protein assay (Thermo Scientific). Protein extracts were fractionated by SDS–polyacrylamide gel electrophoresis, after which they were transferred to a polyvinylidene difluoride (PVDF) membrane and probed with antibodies specific for polη (B-7, Santa Cruz Biotechnology, diluted 1:500), polι (60,668, Novus, diluted 1:1,000), polκ (ab57070, Abcam, diluted 1:1,000), Npm1 (FC82291, Sigma, diluted 1:20,000), FLAG (M2, Sigma, diluted 1:1,000), APE1 (AB194, Abcam, diluted 1:2,000), and tubulin (DM1A, Sigma, diluted 1:20,000). Proteins were visualized using SuperSignal West Pico Chemiluminescent substrate (Pierce). Quantification was done by ImageJ software. Uncropped blots are presented in Supplementary Fig. 9.

### Co-immunoprecipitation

Cells were rinsed twice in cold PBS and lysed in Triton lysis buffer (20 mM Tris-Cl, pH 7.5, 150 mM NaCl, 0.5 mM EDTA, 10% glycerol and 1% Triton X-100) supplemented with protease and phosphatase inhibitors (Sigma) for 1 h at 4 °C. Samples were pre-treated with protein A/G PLUS agarose beads (Santa Cruz Biotechnology), and then incubated with 2 μg rabbit anti polη (H300, Santa Cruz Biotechnology), 2 μg rabbit IgG (Santa Cruz Biotechnology) or 2 μg mouse anti FLAG (M2, Sigma) for 2 h, followed by adding protein A/G PLUS agarose beads or Protein A/G MagBeads (Genscripts) for additional 1 h. Beads were washed three times with lysis buffer, and two additional times with lysis buffer that contained also 250 mM NaCl. Beads were boiled in SDS buffer, and were taken for immunoblot analysis.

### Immunostaining and PLA

The PLA assay was preformed according to the manufacturer’s instructions (OLINK Bioscience). In brief, cells were plated on 12 mm coverslips pre-coated with 10 μg ml^−1^ fibronectin (Sigma) and fixed with 3% paraformaldehyde. When indicated, cells were ultraviolet irradiated with 10 J m^−2^ at time points before fixation. Permeabilization was done with 0.1% Triton X-100. Coverslips were blocked and then probed with rabbit anti polη (AB17725, Abcam, diluted 1:500) and mouse anti-NPM1 (FC82291, Sigma, diluted 1:500). Anti-rabbit-plus and anti-mouse-minus PLA probes were added, ligated and amplified according to the manufacturer’s instructions. Immunostaining of NPM1 was done using anti-NPM1 (FC82291, Sigma, 1:500). Images were captured with Zeiss Axio-Observer Z1 microscope and analysed with CellProfiller software^68^.

### Proteasome or nuclear export inhibition

Proteasomal activity was inhibited by treating MRC5sv or AML cells with 10 μM MG132 (Sigma) for 1–4 h. *NPM1c+* nuclear export was inhibited by treating AML cells with 20 nM Leptomycin B (Santa Cruz Biotechnology) for 3–7 h.

## Author contributions

O.Z., A.Z. and Z.L. designed the research; O.Z. and N.M.-N. performed the research; A.Z., O.Z., E.D., R.N. N.B.-C. and Z.L. analysed the data; U.S., N.E.G., T.C., S.S., C.E.C., M.P.M., R.R., and B.F. contributed new reagents/analytic tools; O.Z., A.Z., R.N., N.E.G., T.C., S.S., C.E.C., M.P.M., B.F., E.D. and Z.L. wrote the manuscript.

## Additional information

**How to cite this article:** Ziv, O. *et al.* Identification of novel DNA-damage tolerance genes reveals regulation of translesion DNA synthesis by nucleophosmin. *Nat. Commun.* 5:5437 doi: 10.1038/ncomms6437 (2014).

## Supplementary Material

Supplementary Information Supplementary Figures 1-9 and Supplementary Tables 1-2

Supplementary Data 1 The results of the TLS-specific screen of 240 genes that were scored as candidate TLS genes in the primary UV sensitivity screen. The information includes for each gene the name, the siRNA library from which it was taken, the effect in the UV sensitivity assay (fold change and P value), the effect in the screen for the TLS assay across a 6-4 photoproduct (fold change, P value, FDR, and number of validated siRNA oligos), and the same for a parallel screen for TLS across a TT-CPD (a major UV DNA damage).

## Figures and Tables

**Table 1:** Summary of the novel TLS genes identified by the 2-stage screen.

**Gene symbol**	**Ultraviolet-sensitivity screen**		**6-4 PP TLS screen**		**CPD TLS screen**		**Examined in MEFs**	**Function category**
	**Fold change**	***P*** **value**	**Fold change**	***P*** **value**	**Fold change**	***P*** **value**		
*ALKBH2*	2.2	1.0 × 10^−5^	1.5	7.9 × 10^−4^	0.9	2.0 × 10^−1^	√	Replication/repair
*DCLRE1A*	1.6	2.3 × 10^−3^	1.7	1.2 × 10^−2^	1.1	4.0 × 10^−1^	√	
*ERCC4*	3.2	<1 × 10^−7^	2.8	1.3 × 10^−5^	1.2	1.8 × 10^−1^	√	
*MCM3*	2.2	<1 × 10^−7^	1.6	6.0 × 10^−3^	1.5	7.0 × 10^−3^	√	
*RUVBL2*	2.7	8.0 × 10^−7^	1.7	1.1 × 10^−3^	1.3	7.5 × 10^−2^	√	
*RPN1*	2.4	<1 × 10^−7^	1.5	3.4 × 10^−3^	1.1	3.2 × 10^−1^		Proteasomal degradation
*SMURF2*	1.6	2.1 × 10^−4^	1.8	8.6 × 10^−6^	1.5	2.8 × 10^−3^		
*UBE2E1*	1.5	1.1 × 10^−3^	3.0	5.3 × 10^−7^	2.5	1.2 × 10^−6^	√	
*UBE2G2*	0.7	1.3 × 10^−3^	1.7	3.4 × 10^−5^	1.1	2.1 × 10^−1^		
*NPM1*	1.7	3.8 × 10^−6^	1.8	4.4 × 10^−3^	1.6	2.9 × 10^−3^	√	Chaperon
*DNAJC6*	1.5	2.4 × 10^−3^	1.7	3.2 × 10^−4^	1.1	2.5 × 10^−1^		
*TRIP11*	1.6	7.5 × 10^−6^	2.5	<1 × 10^−7^	2.2	<1 × 10^−7^	√	Structural Golgi
*VCPIP1*	0.7	1.6 × 10^−3^	1.8	3.3 × 10^−5^	1.3	2.3 × 10^−2^		
*CYLD*	1.6	8.7 × 10^−5^	2.3	1.5 × 10^−4^	1.3	8.3 × 10^−2^	√	Other
*OTUB2*	2.0	1.0 × 10^−7^	1.8	5.6 × 10^−4^	1.4	1.6 × 10^−2^		
*PAPD7*	1.4	2.0 × 10^−2^	1.5	1.0 × 10^−3^	1.1	1.9 × 10^−1^	√	
*SENP2*	1.7	1.3 × 10^−6^	1.1	2.5 × 10^−1^	1.6	2.7 × 10^−3^		

The table lists the 17 novel TLS genes identified by the two-stage screen, divided into functional categories.
