# Supplementary material for: Identification of novel DNA-damage tolerance genes reveals regulation of translesion DNA synthesis by nucleophosmin
Source: Nat Commun. 2014 Nov 25;5:5437. doi: 10.1038/ncomms6437 (PMC4263322; doi:10.1038/ncomms6437)
Supplement: Supplementary Information — Supplementary Figures 1-9 and Supplementary Tables 1-2 [file ncomms6437-s1.pdf]

Supplementary Information

Identification of novel DNA-damage tolerance genes reveals regulation of translesion DNA synthesis by nucleophosmin

Omer Ziv, Amit Zeisel, Nataly Mirlas, Umakanta Swain, Reinat Nevo, Nir Ben-Chetrit, Maria Paola Martelli, Roberta Rossi, Stefan Schiesser, Christine E. Canman, Thomas Carell, Nicholas Geacintov, Brunangelo Falini, Eytan Domany and Zvi Livneh*

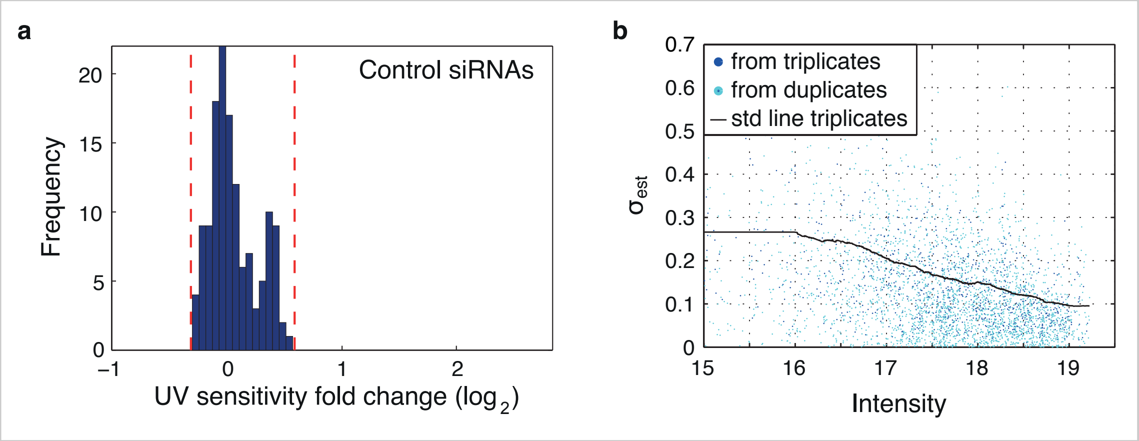

Supplementary Figure 1: Data analysis of the UV sensitivity screen. (**a**) Histogram describing UV-sensitivity fold-changes of the control siRNAs. Dashed red lines correspond to those in Figure 1b. (**b**) Noise estimation for the UV-sensitivity screen. Standard deviation (std) line was calculated using intensity dependent noise model as describe in (Zeisel et al., 2010).

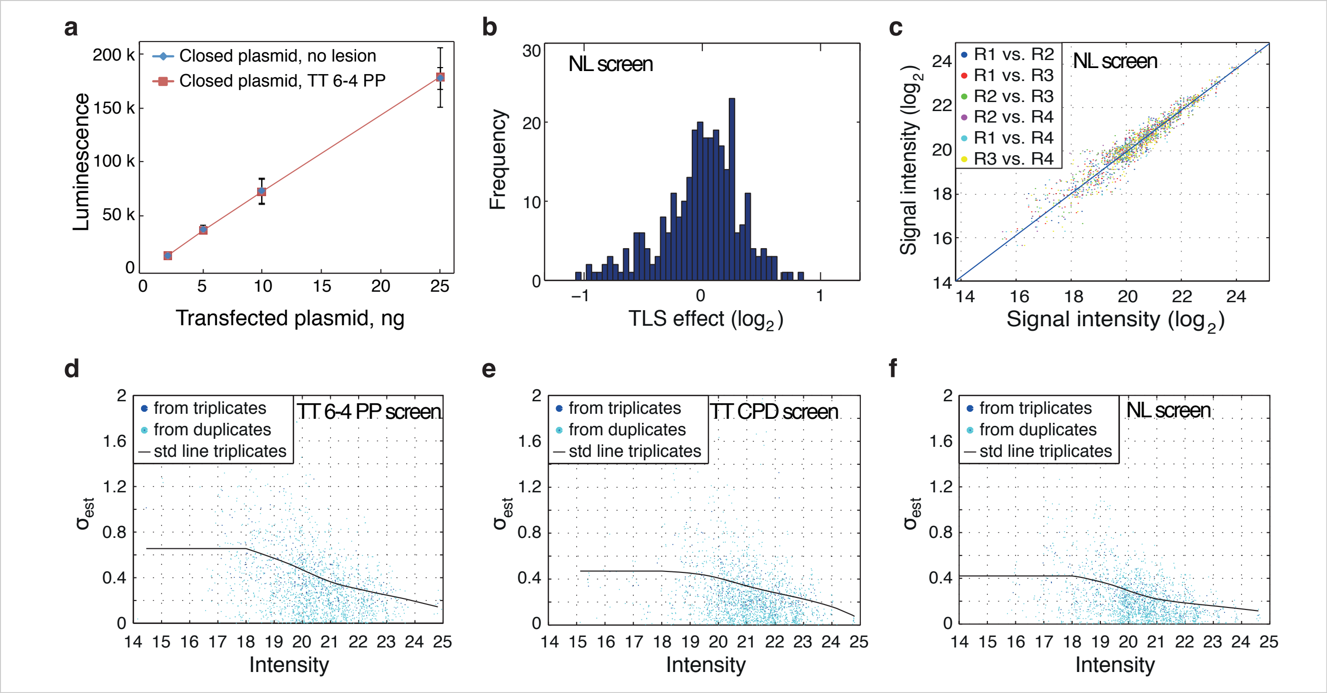

Supplementary Figure 2: Normalization and noise estimation for the TLS screen. (**a**) Luminescent signals from a fully double-stranded Fluc plasmid containing a lesion, and a similar one without the lesion, introduced into *XPA* cells. Values are normalized to control Rluc expressing plasmids. (**b**) Histogram of luminescence values produced by Fluc and Rluc gapped-plasmids without a lesion (No lesion - NL correction), which were introduced into *XPA* cells pre-treated with the siRNA hits from the primary screen. See also Supplementary Experimental Procedures. (**c**) Luminescence values of the four biological replicas plotted against each other from the NL correction described above. (**d-f**) Noise estimation for the TT 6-4 PP TLS screen (**d**), TT CPD TLS screen (**e**), and the NL correction described above (**f**).

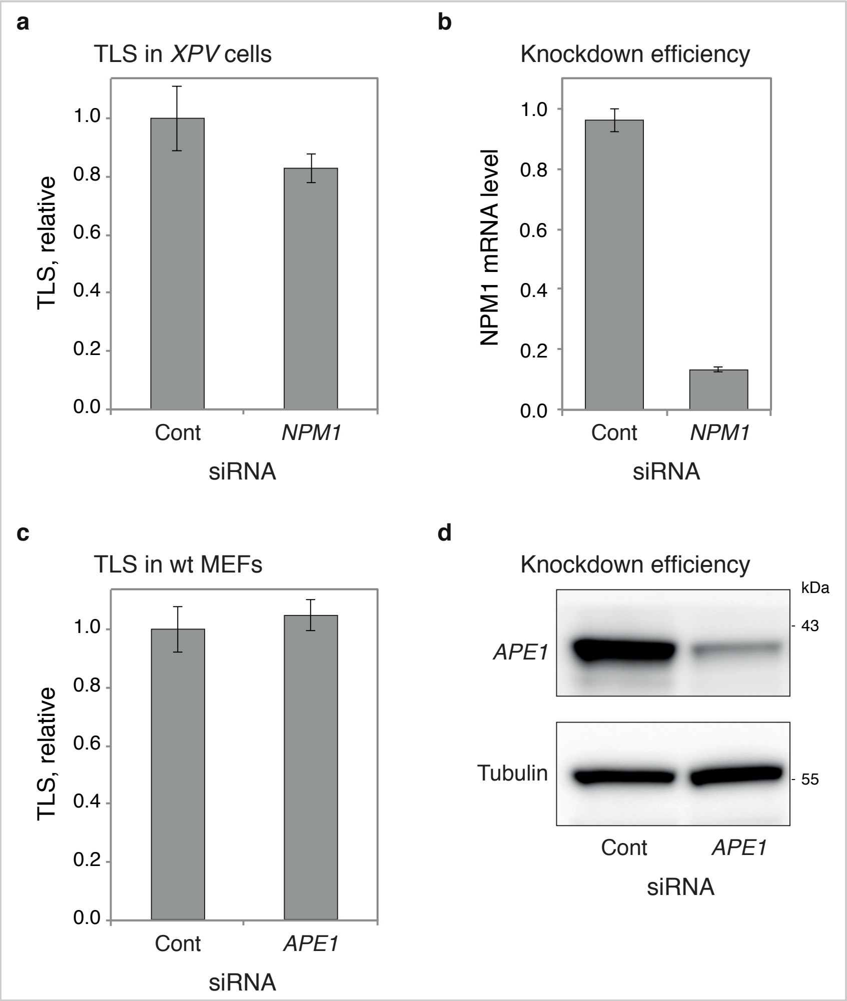

Supplementary Figure 3: NPM1 effect on TLS is mediated via polη and not APE1. (a) Efficiency of TLS across TT CPD, as measured in Polη deficient *PolH*$^{-/-}$ MEF cells pretreated with siRNA against NPM1 or control siRNA. Results of gapped plasmid-based, colony forming TLS assays are presented as the mean ± SE of 3 biological replicas. (b) mRNA knockdown efficiencies in the cells used in (a). Mean values ± s.e.m of 3 replicas are presented. (c) Efficiency of TLS across TT CPD in MEF cells pretreated with siRNA against *Ape1* or control siRNA. Results of gapped plasmid-based, colony forming TLS assays are presented as the mean ± SE of 3 biological replicas. (d) Immunoblot of APE1 extracted from the cells used in (c).

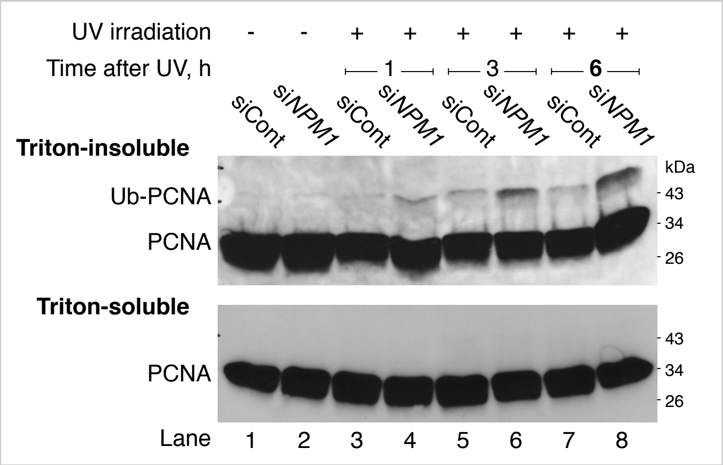

Supplementary Figure 4: PCNA mono-Ubiquitination in *NPM1* knockdown cells. Triton-soluble and triton-insoluble fractions of monoubiquitinated PCNA extracted from MRC5sv cells pretreated with siRNA against *NPM1* or control siRNA.

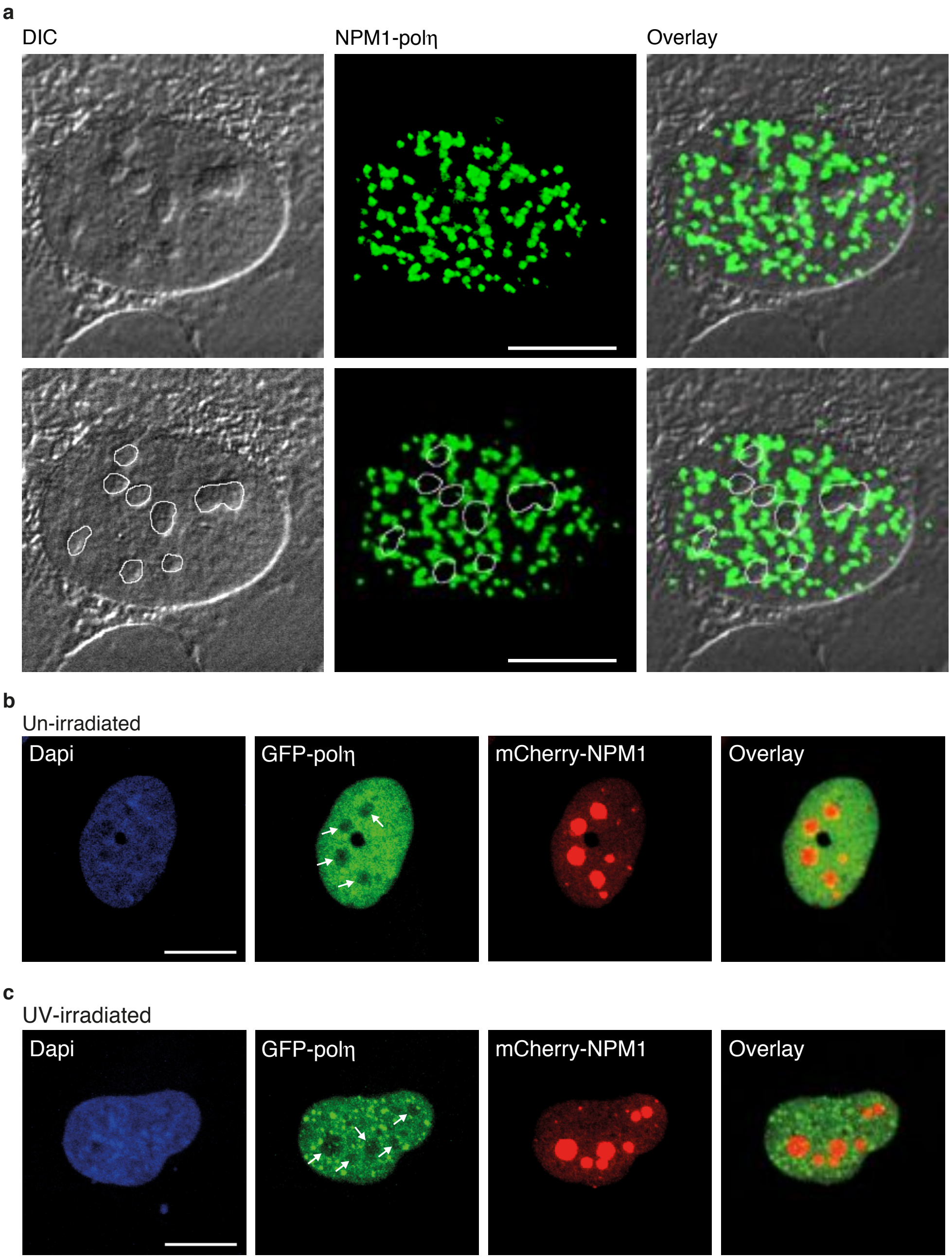

Supplementary Figure 5: Polη interacts with NPM1 in the nucleoplasm. (a) Proximity ligation assay of endogenous polη and NPM1 in MRC5sv cells. Green, polη-NPM1 interaction; white circles mark the nucleoli as determined by the DIC image. White scale bars correspond to 10 µm. (b-c) MRC5sv cells co-expressing GFP-polη and mCherry-NPM1. Unirradiated cells are presented in (b), and UV-irradiated cells in (c). White arrows represent the nucleoli as indicated by strong NPM1 staining. White scale bars correspond to 10 µm.

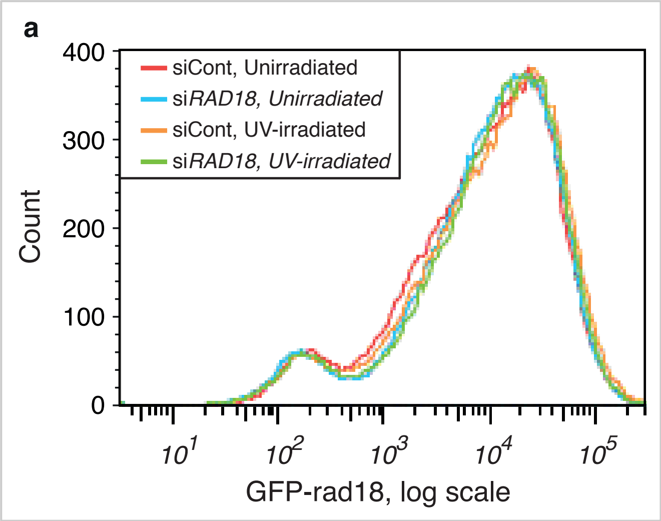

Supplementary Figure 6: FACS analysis of MRC5sv cells stably expressing GFP-RAD18 and transfected with siRNA targeting NPM1.

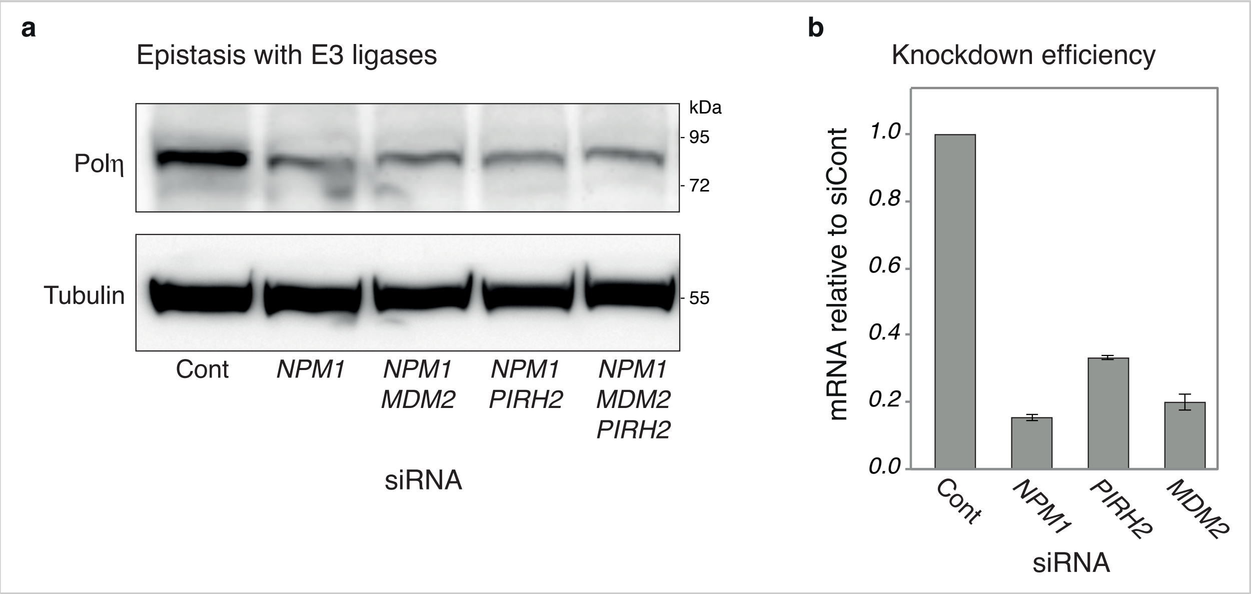

Supplementary Figure 7: Epistasis analysis of NPM1 and the E3 ligases PIRH2 and MDM2. (a) Immunoblot of polη extracted from MRC5sv cells pretreated with siRNA against the indicated genes. (b) mRNA knockdown efficiencies in the cells used in (a). Mean values ± s.e.m of 3 replicas are presented.

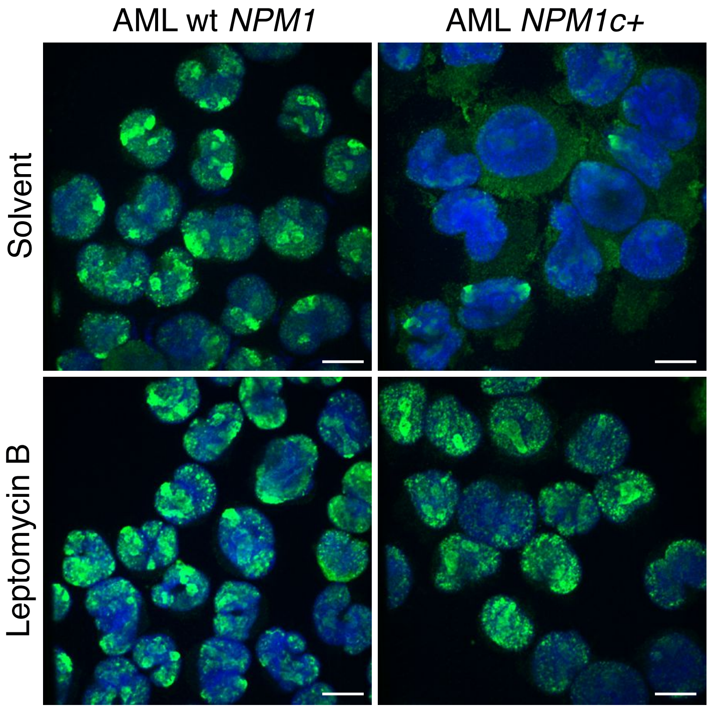

Supplementary Figure 8: Immunostaining of NPM1 in AML2 and AML3 cells pretreated with Leptomycin B, or its solvent as a control. Blue, DAPI staining of the DNA in the nucleus; Green, staining of NPM1. White scale bars correspond to 10 μm.

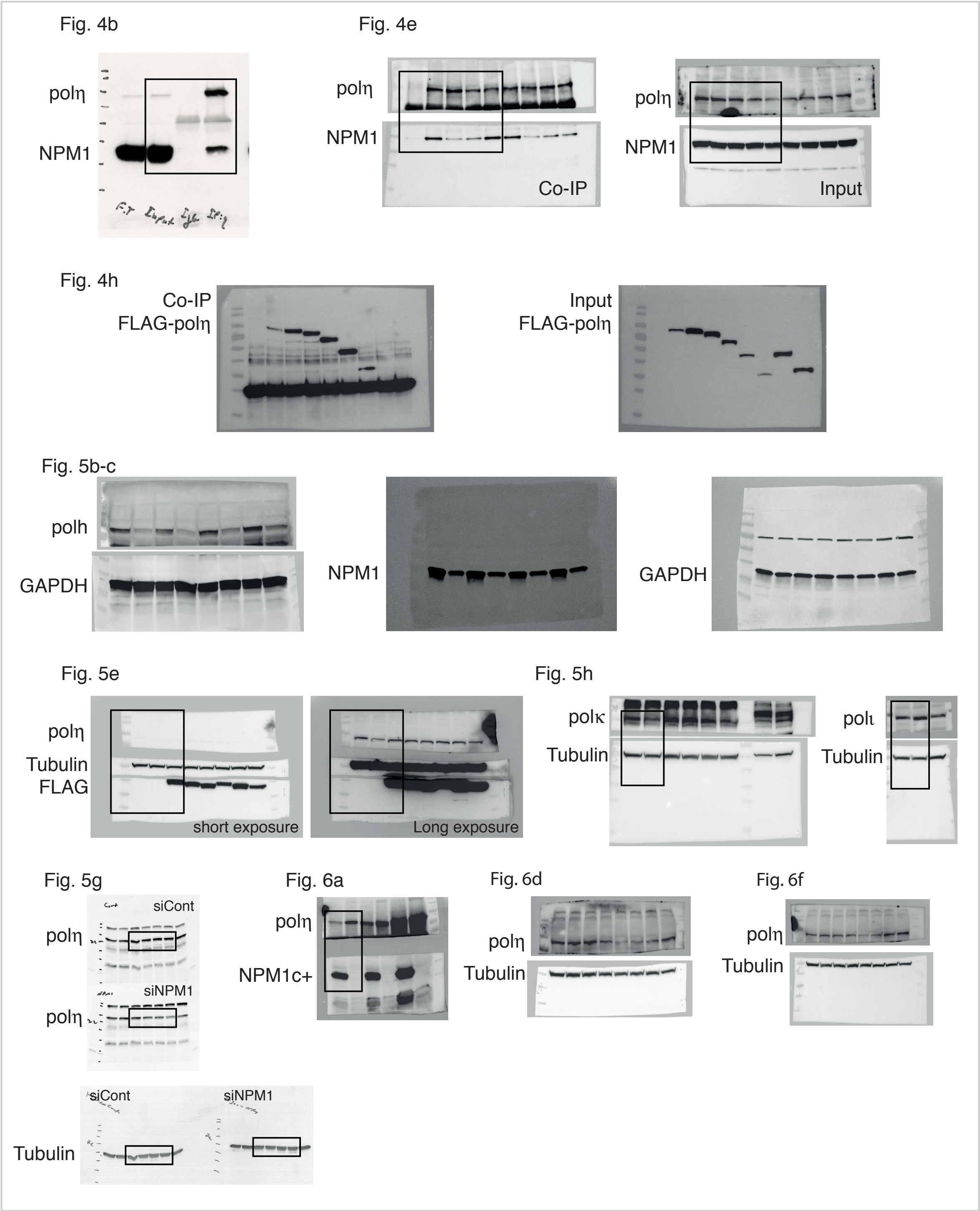

Supplementary Figure 9: Non-cropped gel images for relevant figures in the manuscript.

Supplementary Table 1. siRNA libraries representation within the screen hits

siRNA library	Total examined	UV screen		TLS screen		Novel TLS candidates
		**Right side effect**	**Left side effect**	**Right side effect**	**Left side effect**	
DNA Repair	284	72 (25%)	4 (1%)	24 (8%)	2 (<1%)	6 (2%)
Ub Ligases	241	42 (17%)	15 (6%)	9 (4%)	2 (<1%)	3 (1%)
DUBs	125	20 (16%)	12 (10%)	10 (8%)	2 (2%)	4 (3%)
Cancer	301	37 (12%)	17 (6%)	12 (4%)	1 (<1%)	4 (1%)
Cell Cycle	111	42 (38%)	2 (2%)	11 (10%)	0 (0%)	1 (<1%)
Total	1062	213 (20%)	50 (5%)	66 (6%)	9 (<1%)	18 (1.7%)
Non redundant		**192**	**45**	**52**	**7**	**17**

Representation of the siRNA libraries used for the screen within the hits of the primary and secondary screens. Right side effects denote elevation in UV sensitivity or reduced TLS capability, while left side effects denote reduced sensitivity to UV or enhanced TLS capability. ‘Novel TLS candidates’ were confirmed with 2-4 different siRNA oligonucleotides.

Supplementary Table 2. Enrichment of DNA repair/ tolerance pathways within the screen hits

Pathway	Scored	Not scored
TLS	POLH, POLK, REV1, REV3L, REV7, RAD6A, USP1, MMS2, UBC13, ATR, CHEK1, RAD17	POLI, RAD6B, RAD18, P21, P53, DDB1, HSP90A, HSP90B
HRR	ERCC4, RAD52	BLM, BRCA1, BRCA2, DMC1, EME1, EXO1, MRE11A, MUS81, NBS1, PIR51, RAD50, RAD51, RAD51B, RAD51C, RAD51D, RAD54B, RAD54L, RECQL4, RECQL5, TOP3A, WRN, XRCC2, XRCC3
NHEJ	-	ARTEMIS, DNA-PKcs, KU70, KU80, LIG4, MRE11A, NBS1, POLL, POLM, RAD50, XRCC4
NER	ERCC4	CCNH, CDK7, CETN2, DDB1, DDB2, ERCC1, ERCC2, ERCC3, ERCC5, ERCC6, ERCC8, GTF2H1, GTF2H2, GTF2H3, GTF2H4, GTF2H5, LIG1, MMS19, MNAT1, RAD23A, RAD23B, RPA1, RPA2, RPA3, XAB2, XPA, XPC
MMR	-	MSH2, MSH3, MSH4, MSH5, MSH6, PMS1, PMS2, PMS2L3, PMS2L4
BER	-	APEX1, APEX2, APTX, FEN1 HUS1, LIG3, MBD4, MPG, MUTYH, NEIL1, NEIL2, NEIL3, NEIL3, NTHL1, OGG1, PARP1, PARP2, PARP3, PNKP, SMUG1, TDG, UNG, XRCC1
FA	-	BRCA2, BRIP, FANCA, FANCB, FANCC, FANCD2, FANCE, FANCF, FANCG, FANCL, RAD51C
ATR	ATR, CHEK1, RAD17	BRCA1, CLSPN, HUS1, NBS1, RAD1, RAD9A, RAD9B
ATM	TP53BP1	ATM, BRCA1, CHEK2, FANCD2, MRE11A, NBS1, RAD50, H2AFX, MDC1

The table lists known DNA repair and tolerance genes that were present in the siRNA libraries, and whether they were scored as hits. A high selectivity for TLS genes is observed, and quantified in Figure 2h.

Supplementary Table 3. siRNA sequences in the siRNA pools used in this study

Target gene	siRNA #1	siRNA #2	siRNA #3	siRNA #4
h*NPM1*	GAGCACCAGU<br>UAUCUUUAA	CUAAAGGCC<br>GACAAAGAU	ACUUUAAGGU<br>GGAUAAUGA	CGAAGGCAGU<br>CCAAUUAAA
m*NPM1*	GUGGAAGCCA<br>AGUUCAUUA	GCACAUUAG<br>UGGACAGCU	CCAGGAGGCU<br>AUUCAAGAU	GAAAAUGUCU<br>GUACAACCA
m*PAPD7*	GCAGAUAUUU<br>GGCAGCUUU	GAAAUGAUG<br>UUGGACGGG	UAGGAAGAAU<br>CAUCAAAGU	GCUACAGUGC<br>CCAUCAUAA
m*RUVBL2*	GAAGACAGCC<br>AUUGCCAUG	CACAGUACA<br>UGAAGGAGA	GCUCAAAGGC<br>GAAACAAUG	UGACAAGAAU<br>CGAGCGAAU
mCYLD	GGACAUGGAU<br>AACCCUAUU	CAACUGGGA<br>UGGAAGGUU	GAUAGUCAAU<br>CCUCUGAGA	CAAUUCAGCA<br>GUUAUUAGA
m*TRIP11*	CCAAAGACGA<br>GGUCGGCAA	GAGAAGAAC<br>ACCUCGCUAA	AGAAUGAGGU<br>CCAGCGCUU	GUGACUAUGA<br>AGAGCGUAU
m*MCM3*	GAAGGCAGCU<br>UUGGCUCAA	CAAAGUCGU<br>UCGCAGUGU	GCAAGAUACC<br>CGCAUUUAU	CUACUAAGAA<br>GACCAUAGA
m*DCLRE1A*	GCAAAUGGAU<br>AUAGGCGUG	GCUAUGUAC<br>UUCCACAAU	ACUUAGAAAU<br>GAAGCGUUU	GCACUUACUG<br>CAUCGGAAA
m*ABH2*	GUACAAAGAU<br>GGUUGCGAC	GAUCGAGUC<br>UGCGAGGUA	GGUCUUACCC<br>UGACACCAA	CCAGGAAGCA<br>GGCGACCUA
m*UBE2E1*	GAGUGGAGAU<br>CAACCAUUC	GGUGGAGUA<br>UUCUUCCUG	GCAAUCCUGC<br>UGACCCUUU	CUUUAGACCC<br>UCCGCCAAA
m*ERCC4*	GGAGCGUGCU<br>UCCGCCAAA	GCCUGAAGU<br>UGUAGAGAU	UCACAACCCG<br>UCACUUGAA	GCCGAAUACU<br>CGUGGUUGA
h*PIRH2*	GGAGACAGCU<br>GGAUGAUGA	CUAGAUCGC<br>UUUAAAGUA	GCACAUUGUU<br>UGGAGAAUA	UAACAAUACC<br>CUUACGAAU
h*MDM2*	GCCAGUAUAU<br>UAUGACUAA	GAUGAGAAG<br>CAACAACAA	CCCUAGGAAU<br>UUAGACAAC	AAAGUCUGUU<br>GGUGCACAA

All siRNAs were purchased from Dharmacon. ‘h’ and ‘m’ before the target gene stands for human and mouse sequences, respectively.

Supplementary Table 4. qPCR primers used in this study

Target	Forward primer (5' to 3')	Reverse primer (5' to 3')
Mouse *Cyld*	TGGAGTACCCACAATTCAGCA	CCGAGGCATCTGGATAATCA
Mouse *Npm1*	CTCGATGGATATGGACATGAGTC	TTTGTCAGCCTTTAGTTCACAGC
Mouse *Trip11*	TGAGTCTGAACTTGGACATTGG	CAGAGCTTTGTGCTCCCTGT
Mouse *RuvBL2*	ATCATTGAAGGGGAGGTGGT	CGTGGTCTTGAGGGTCAGTT
Mouse *Ube2E1*	TCGAAGCTTCTCTCCACCAG	GGCGGAGGGTCTAAAGTGAT
Mouse *Ercc4*	TGCCTTTGACACTGGCTTTT	TTCAGGCTTGTGTTGTTCCA
Mouse *Mcm3*	CCGTGGAGCTGGTTCAGTAT	TGTCTTCCTGGCTCTTTTCCT
Mouse *PolS*	AAAGCCATGACCAGTGGGTA	AGGAACTCCGTCCAACATCA
Mouse *AlkBH2*	CCGAGAGTTGGAGCAAGAAG	CACTTTCCGAACACCTGGAC
Mouse *Dclre1A*	TGCGACTCTTTGGTTCACCT	GAACGCCAAAATCTGATCGT
Mouse *Rev3L*	AATGCATCACATGCAACTGG	GGGTTCAACACCTTCCAACA
Human *NPM1*	ACTCCTAAAACACCAAAAGGACC	TTCCACTTTGGGAAGAGAACC
Human *PIRH2*	AGCGGTCAAGAGCGAGGT	AAAGCTTGTCACAGCAAGGTG
Human *MDM2*	AGAAACCTTCATCTTCACATTTGG	GGGGGATTCATTTCATTGC

All primers were designed using the Primer3 web interface, and were pre-examined for linearity and specificity.
